# Synthesis of Recommendations From 25 Countries and 31 Oncology Societies: How to Navigate Through Covid-19 Labyrinth

**DOI:** 10.3389/fonc.2020.575148

**Published:** 2020-11-19

**Authors:** Konstantinos Kamposioras, Davide Mauri, Konstantinos Papadimitriou, Alan Anthoney, Nadia Hindi, Branka Petricevic, Mario Dambrosio, Antonis Valachis, Pantelis Kountourakis, Jindrich Kopecky, Cvetka Grašič Kuhar, Lazar Popovic, Nataliya P. Chilingirova, George Zarkavelis, Ramon Andrade de Mello, Natalija Dedić Plavetić, Christos Christopoulos, Bianca Mostert, John R. Goffin, Dimitiros Tzachanis, Haytham Hamed Saraireh, Fei Ma, Ida Pavese, Maria Tolia

**Affiliations:** ^1^Department of Medical Oncology, The Christie NHS Foundation Trust, Manchester, United Kingdom; ^2^Department of Medical Oncology, University Hospital of Ioannina, Ioannina, Greece; ^3^Early Clinical Trials Unit, Antwerp University Hospital, Antwerp, Belgium; ^4^Leeds Institute of Medical Research at St James’ Hospital, University of Leeds, Leeds, United Kingdom; ^5^Department of Medical Oncology, University Hospital Virgen del Rocío, Sevilla, Spain; ^6^TERABIS Group, IBiS (Instituto de Biomedicina de Sevilla)/HUVR/CSIC/Universidad deSevilla), Sevilla, Spain; ^7^Medizinische Abteilung, Zentrum für Onkologie und Hämatologie mit Ambulanz und alliativstation Wilhelminenspital, Vienna, Austria; ^8^Department of Clinical Oncology, Clinica San Carlo, Milan, Italy; ^9^Department of Oncology, Faculty of Medicine & Health, Örebro University, Örebro, Sweden; ^10^Medical Oncology Department, Bank of Cyprus Oncology Centre, Nicosia, Cyprus; ^11^Department of Clinical Oncology, University Hospital, Charles University—Faculty of Medicine in Hradec Kralove, Hradec Kralove, Czechia; ^12^Department of Medical Oncology, Institute of Oncology Ljubljana, Ljubljana, Slovenia; ^13^Oncology Institute of Vojvodina, University of Novi Sad, Novi Sad, Serbia; ^14^University Specialized Hospital for Active Treatment in Oncology, Medical Oncology Clinic, Sofia, Bulgaria; ^15^Medical University Pleven, Pleven, Bulgaria; ^16^Escola Paulista de Medicina, Federal University of São Paulo, São Paulo, Brazil; ^17^Department of Biomedical Sciences and Medicine, University of Algarve, Faro, Portugal; ^18^University Hospital Centre, Zagreb Department of Oncology, School of Medicine, University of Zagreb, Zagreb, Croatia; ^19^Service de Radiothérapie Oncologique, Groupe Hospitalier Intercommunal Le Raincy-Montfermeil, Montfermeil, France; ^20^Department of Medical Oncology, Erasmus MC Cancer Institute, Rotterdam, Netherlands; ^21^Department of Oncology, McMaster University Juravinski Cancer Centre, Hamilton, ON, Canada; ^22^Department of Medicine, University of California San Diego School of Medicine, La Jolla, CA, United States; ^23^Radiation Oncology Department, Jordanian Royal Medical Services, Amman, Jordan; ^24^Department of Medical Oncology, National Cancer Center/National Clinical Research Center for Cancer/Cancer Hospital, Chinese Academy of Medical Sciences and Peking Union Medical College, Beijing, China; ^25^Service d’Oncologie, GHT Grand Paris Nord-Est, Montfermeil, France; ^26^Department of Radiotherapy, School of Medicine, University of Crete, Heraklion, Greece

**Keywords:** Covid-19, recommendations, international, oncology, societies

## Abstract

**Introduction:**

Pandemic COVID-19 is an unexpected challenge for the oncological community, indicating potential detrimental effects on cancer patients. Our aim was to summarize the converging key points providing a general guidance in order to support decision making, pertaining to the oncologic care in the middle of a global outbreak.

**Methods:**

We did an international online search in twenty five countries that have managed a surge in cancer patient numbers. We collected the recommendations from thirty one medical oncology societies.

**Results:**

By synthesizing guidelines for a) oncology service delivery adjustments, b) general and specific treatment adaptations, and c) discrepancies from guidelines comparison, we present a clinical synopsis with the forty more crucial statements. A Covid-19 risk stratification base was also created in order to obtain a quick, objective patient assessment and a risk-benefit evaluation on a case-by-case basis.

**Conclusions:**

In an attempt to face these complex needs and due to limited understanding of COVID-19, a variability of recommendations based on general epidemiological and infectious disease principles rather than definite cancer-related evidence has evolved. Additionally, the absence of an effective treatment or vaccine requires the development of cancer management guidance, capitalizing on comprehensive COVID-19 oncology experience globally.

## Introduction

The rapid international spread of COVID-19,-linked to the severe adult respiratory syndrome SARS-CoV-2- along with the proliferation of severe morbidity cases, often leading to death, has placed extreme pressure on health care systems, necessitating global coordination and collaboration between governments, healthcare professionals and organizations. Despite our poor understanding of this new coronavirus, recommendations for the management of specific patient groups were rapidly developed.

Preliminary reports identified the high risk of cancer patients contracting COVID-19 and having a worse outcome than the general population (1, 2). Cancer alone was associated with a potential intensive care unit admission and death risk (OR 5.4, 95% CI 1.8–16.2) (1). This led to a rapid evolution of patients’ and clinicians’ guidance from national and international oncology societies (3). The scientific evidence was- and is still- missing though, and in many cases, this guidance was based on extrapolation of information from previous pandemics, regional guidance and logical judgements (4, 5).

Herein, we summarize the guidance provided so far from medical oncology societies to allow a better interpretation and implementation of proposed actions to face the evolution of the COVID19 pandemic.

## Methods

An established collaborative group of oncologists (6) provided guidelines and recommendations from their national medical societies for cancer management during COVID-19 and present patients’ expectations from oncology societies, respectively. Guidelines from individual institutes were not included, unless it was the only cancer centre in the country, while oncology-related state guidance was captured as well. Documents which were exclusively patient-facing (i.e., did not provide guidance to healthcare providers) were also excluded.

A typical medical literature search was not conducted due to the sudden nature of the pandemic, the rapid and recent production of the documents, and their frequent publication outside of the medical literature (as for example on agency websites). For reasons of urgency, the available documents were not produced using accepted guideline protocols, such as AGREE II, and thus were not graded.

Results were summarized and compared by type of recommendation (preventive, intervention, and treatment measures), risk group and type of malignancy when applicable, with focus on converging points, discrepancies, potential shortcomings, and underlying evidence level.

## Results

Medical oncology recommendations from 25 countries and 31 international organizations were analyzed (**Appendix Table 1**). Oncology societies provided general and specific instructions to revise cancer patient’s service delivery and treatment. The development of a clear risk minimization strategy was common, focusing on patients’ social contacting reduction. Below, we present the converging points from different societies classified in: a) guidelines for oncology service delivery adjustments, b) general and specific treatment adjustments, c) special issues guide, d) discrepancies from guidelines comparison.

### Service Delivery

Most societies agree on minimization of: hospital visits, unavoidable visits duration and waiting areas overcrowding. Service delivery recommendations include physical attendance only when essential and substitution with “tele”-service. Similar suggestions include: treatment prioritization strategies; stratified follow-up models; follow-up visit postponement and use of tele-consulting; slot appointment planning; waiting for appointments in the car or other non-clinical areas; no escorts in clinical facilities, unless essential (**Appendix Table 2**).

Furthermore, phone triage the day before treatment (as well as before entering the facilities in checkpoint areas), waiting room rearrangements with respect to social distancing and creation of a separate circuit for the oncological patients are commonly proposed as measures to further reduce the risk of transmission (**Appendix Table 2**).

Other recommendations include staff training to triage and test patients, to isolate positive cases and use Personal Protective Equipment, as well as patient guidance in preventive measures and symptoms report (**Appendix Table 2**). Ways to involve infected/isolated healthcare practitioners in service delivery are also proposed: virtual MDT attendance, telephone/video consultations (especially for follow-ups), identification of vulnerable patients, and patients suitable for remote monitoring/follow-up and data entry (7).

Reduced mobility and physical contacts of medical staff within hospitals are also recommended. MDTs as well as other meetings organization *via* video-applications is also advised; similarly, ward rounds restriction to two doctors is recommended (8).

### Medical Treatment: General Considerations

Case by case decisions are proposed, and the continuation of anti-cancer treatment should be individualized according to the patient’s needs. Most societies agree on an upfront risk/benefit discussion with the patient regarding treatment continuation. SEOM recommends that it be documented in the informed consent, with a clear description of the potential risks. MDT discussion with the patient on cancer treatment adjustments is advisable (7).

A substantial limitation of physical contacts is a key point, leading to several treatment adjustments, such as: treatments delays/breaks when clinically appropriate or after discussion with the patient; home blood specimen collection before treatment; home deliveries of long-term treatment supplies and therapies for low risk injectable and oral agents; tele-monitoring or repetitive treatment; treatment interval increase by limiting the use of dose-dense chemotherapy regimens and adjustment to the longest cycle regimens possible; whenever applicable, change i.v. chemotherapy to oral or subcutaneous forms. Supportive (e.g.bisphoshonates) treatments could be delayed and blood transfusions limited to the absolutely necessary (**Appendix Table 3**).

Aiming at immunosuppression risk reduction, many societies [MOGA/AGCA, Bulgarian, Chinese, HDIO, IOL, SEOM, SOF/RCCC, NHS/NICE, ASCO/NCCN/ACS/CDC, ESMO] recommend the use of prophylactic GCSF +/- antibiotics (2, 9–12) and also reducing or tapering steroids as anti-emetics or immune-suppressants when appropriate. Dutch Oncology Society (NVMO) is against the prophylactic use of GCSF +/- antibiotics (13). SEOM suggests avoiding initiation of immunosuppressive treatment in possibly infected patients, with a history of contacts at risk or when 2-3 weeks delay is not health-threatening; the plan can be reviewed every 2–3 weeks by phone. BSMO suggests to critically review the urgency for initiation of new cancer therapy and consider postponement for several months; if 12 weeks postponement is not feasible, contacts should go on as pre-planned (14).

In order to facilitate these decisions and prevent staff shortage or infrastructure capacity overload at a potential second pandemic outbreak, risk assessments and treatment prioritization models were proposed by several societies (7, 15, 16) and are presented in **Table 1**.

**Table 1:** Risk assessments and treatment prioritization models.

Priority group	ESMO	NICE	HPSP	HeSMO
**1**	Life threatening conditions, clinically unstable pts or when benefit is higher than risk in terms of survival or QoL (high priority group)	Curative therapy with a high (>50%) chance of success	Pts treated with curative intent: **≤**60 y old or life exp ≥5 y, or both	Pts with imminent life threat: e.g., metastatic germ cell tumors, aggressive neoplasms
**2**	Pts in non-critical status, whose oncological treatment benefit qualifies for intermediate priority: treatment should not be delayed > 6 weeks as this could be detrimental on the oncological outcome	Curative therapy with an intermediate (15–50%) chance of success	Pts treated with non-curative intent: **≤**60 y old or life exp ≥5 y, or both, and under early line of treatment	Pts with severe QoL deterioration due to cancer symptoms and high morbidity
**3**	Pts stable enough for their treatment to be delayed for the duration of the pandemic and/or when the intervention benefit is minimal: no survival gain with no change nor reduced QoL (low priority group)	Non-curative therapy with a high (>50%) chance of >1 yr life extension	Pts treated with non-curative intent: those under PD or when treatment interruption can be life threatening	Pts undergoing therapy with curative intent
**4**		Curative therapy with low (0–15%) chance of success OR non-curative therapy with an intermediate (15–50%) chance of > 1 y life extension		Pts under palliative therapy but with significant survival benefit
**5**		Non-curative therapy with a high (>50%) chance of palliation/temporary tumor control but < 1 year life extension		Pts under palliative therapy with modest survival benefit with/or significant symptoms control
**6**		Non-curative therapy with an intermediate (15–50%) chance of palliation and temporary tumor control with < 1 y life extension.		Pts under palliative therapy without survival benefit or symptom control
**7**				*Supportive measures or cases where therapy does not affect patient outcome*

Pts, patients; exp, expectancy, y, year.

### Setting Specific Management

Certain societies provide specific treatment adjustments guidance and additional detailed considerations by cancer type and treatment setting. ESMO recently published detailed management guidelines stratified by risk priority group- as described above- and type/stage of malignancy (16).

#### Invasive Procedures

The decision for the necessity for interventional radiology procedures and diagnostic biopsies depends on its importance in guiding subsequent treatment decisions and on the patient’s co-morbidity. Curative primary tumour resections should not be postponed or omitted. Metastasectomies or debulking surgeries should be performed upon personalized risk-benefit evaluation. A surgical procedure that could postpone (neo) adjuvant chemotherapy should be considered.

#### Adjuvant and Neoadjuvant Treatment

As long as the goal is the cure, (neo)-adjuvant chemotherapy or immunotherapy should not be postponed or omitted. However, when the benefit is considered marginal, the risk for fatal coronavirus infection should be weighed against the potential benefit. Use of regimens with the longest cycle possible is suggested. Adjuvant hormonal treatment should be continued.

#### Treatment for Metastatic Disease

Continuation of intravenous chemotherapy with or without immunotherapy should be individualized. Maintenance therapy could potentially be discontinued and treatment holidays should be offered whenever appropriate. Palliative and late line systemic treatments should be probably postponed or adapted to the longest cycle regimen possible or switch to oral regimens. Some agencies, i.e., CCO, have provided detailed priority lists to facilitate treatment decisions by disease site.

Immunotherapy should be continued on basis of risk benefit equation, with increased alertness for respiratory infection symptoms. Hormone therapy treatment should be continued.

Treatment with CDK4/6 inhibitors should be re-evaluated and discontinued if potential benefit is low or adjusted according to immunosuppression risk. The benefit of mTOR inhibitors should be evaluated on a case-by-case basis given the risk for pneumonitis.

### Patients With Confirmed COVID-19 Infection: When to Go Back on Treatment?

There is limited evidence on the link between recent oncological treatment and severe COVID-19 events (1, 17). It is also unclear when to restart treatment after COVID-19 infection. ASCO recommends to hold treatment until the patient is asymptomatic or there is proof of infection resolution, but in cases of severe cancer complication risk, restarting therapy is advised. NICE suggests restarting treatment after one negative SARS-Cov-2 test (7). Finally, French guidance recommends treatment continuation after patient’s recovery (15). To date, ESMO issued only a short statement suggesting for treatment initiation or continuation for SARS-CoV2-positive cancer patients if they are a- or pauci-symptomatic, still fit to be treated and after proper risk/benefit analysis.

Furthermore, according to CDC, severely immunocompromised patients, after COVID-19 infection, can discontinue transmission-based precautions after at least two negative consecutive nasopharyngeal swab specimens collected ≥24 h apart, when accompanied by fever resolution (without medication) and respiratory symptoms improvement (18); according to ASCO, it would be reasonable to initiate/resume anti-cancer therapy, once transmission-based precautions are no longer necessary. Still, given the limited data and the ongoing research, further updates on this issue are expected.

### Special Issues

#### Senior Patients

No specific guidance could be identified for this patient group, with an approach proportional to the general population and based on a documented risk assessment. ESMO suggests “more intensive” surveillance, especially with co-morbidity, but without specific recommendation.

#### Supportive Management

Patients on supportive management or end-of-life treatment should be managed like the general population, any investigations with no symptom control provision should be avoided and hospitalization should be considered when needed (15). ESMO provides a detailed prioritization list. An at-home service is preferable, while admission should be offered in intensive interventions, especially in oncologic emergencies (e.g., spinal cord compression, severe pain, etc.).

#### Clinical Trials

FDA and SEOM provide guidance on clinical trials conduction during the pandemic, with focus on participants’ safety (14). The continuation of an investigational product depends on individual circumstances. Patients under treatment should limit per protocol-specified visits. Alternative ways to assess/follow up patients are recommended after contacting with CRAs/sponsors (e.g., phone contact, virtual visit, alternative location for assessment, including local community labs or imaging centres). Inclusion of new patients should be considered on a case-by-case basis (19).

SEOM suggested that protocol’s recommendations should be strictly followed with dose delays and adjustments as per protocol but adapted to the healthcare and epidemiological situation. BSMO suggested a continuation of ongoing trials, without on-site monitor visits, but no new trials initiation (14). The Dutch and NICE recommendations suggested to continue treatment for patients already included within trials, but to stop recruitment (7, 8). ESMO supports treatment continuation within a clinical trial, provided that benefits outweigh risks, with a possible adaptation of procedures without affecting patient’s safety and study conduct.

#### Ventilator Support and Resuscitation Status

The oncology treating team must inform the intensivist physicians (ICU) on the need for intubation and ventilation. A detailed description of the oncological status is crucial when curative treatment is given (20). The Dutch recommendations suggest to proactively discuss with the patients about the escalation level, resuscitation status (DNR) and ventilation strict policies (8).

#### Well-Being and Emotional Resilience

ESMO and ASCO have addressed the importance of healthcare providers’ mental well-being, with ASCO providing specific suggestions on their mental health.

#### Web and Media

Reliable information from scientifically driven sources should be followed, while using or sharing social media accounts and rumours should be critically appraised (16).

### Ethics

Expectations that the pandemic may constrain resources has led to the recommendation of care prioritization based on expected outcomes. The ethical framework has been provided by some documents, providing support for communication with patients and families when limitations are required [CCO, BCC].

### Discrepancies Between the Guidelines

#### Bone Marrow Growth Factor Therapy

Generally, guidelines support the use of prophylactic G-CSF in order to decrease the risk of hospital admission due to neutropenic sepsis that could expose patients to Covid-19 infection and divert healthcare resources (**Appendix Table 3**) (2, 9–13). However, Dutch guide recommends against G-CSF use given the unclear impact on COVID-19-infection and potential increase of risk for acute respiratory distress syndrome (14). Further research is required to determine the effect of G-CSF during Covid-19 (2, 9–14).

#### Immunotherapy

The risks for patients on immune checkpoint inhibitors and CTLA4 antagonists from COVID-19 infection are also uncertain as the significance of the resulting immune effect on clinical outcome is currently debatable (2, 20–23). Still, their potential toxicity profile, including respiratory morbidity creates concerns. According to ESMO, close monitoring for specific symptoms, e.g., pneumonitis or infection, is recommended, to allow prompt withdrawal of treatment and possible referral to COVID-19 diagnostic pathway. ASCO gives no guide for immunotherapy since no reliable evidence is available and suggests literature follow-up.

#### Personal Protective Equipment (PPE)

Face mask use has been a conflicting point, but now there is mostly a consensus on mask use both by medical staff, patients and relatives during hospital visits See also **Appendix Table 4**.

## Discussion

The COVID-19 pandemic has led to the adaptation of new clinical strategies aiming at physical contacts minimization and adoption of tele-practising models. The oncologists’ challenge is to provide effective treatment and support cancer patients. A plethora of clinical practice guidances has rapidly developed, agreeing on the main changes to be implemented.

The oncologic society’s first priority has been the implementation of an upfront strategy based on safety and treatment efficacy. However, strategic measures derive from general epidemiologic and infectious disease knowledge from prior epidemics (4, 5), which is still poor in the case of COVID-19. Yet, continuous research begins to offer a better understanding of the new coronavirus (24–26). Due to the lack of definitive evidence on the COVID-19 exact pathological profile, guidelines by different societies may vary in specific areas, with one example being the controversy on face mask use, at least until recently (27).

Even more challenging has been the guidance about the use of G-CSF (2, 9–13). Potential benefits may arise from reducing the likelihood of hospital admission due to chemotherapy induced neutropenic fever or sepsis. However, neutrophilia and neutrophil-to-lymphocyte ratios predict poor outcomes in patients with COVID-19 (11, 28). Neutrophilia could be a source of excess neutrophil extracellular traps (NETs); the formation of which can drive a variety of severe pathologies in the lungs, induce mucus accumulation in airways and drive ARDS (29). NETs are also implicated in the development of arterial and venous thrombosis, a feature observed in individuals with severe COVID-19 infection (30). Furthermore, severe COVID-19 is associated with increased plasma concentrations of pro-inflammatory cytokines (cytokine storm) and other molecules including G-CSF (31, 32). Taken together, these data generate uncertainty on the risk/benefit balance concerning the use of G-CSF (2, 9–13).

Whereas many of the recommendations for cancer treatment relate to cytotoxic chemotherapy, guidance on newer therapies, e.g., immune checkpoint CDK4/6, mTOR, and PARP inhibitors, appears only intermittently. For these treatments, we are only based on basic clinical science to help predict possible consequences of COVID-19 infection. Additionally, some of these treatments have adverse effects, like pneumonitis or severe myocarditis, that simulate symptoms arising from COVID-19 infection, posing a threat to appropriate clinical management and possibly compromising survival (33). Currently, most society guidelines suggest a continuation of such treatments upon individualized risk/benefit assessment, but scientific validation is awaited.

It should be emphasized that currently there is a significant lack of specific guidance required for patients with, or recovering from COVID-19 and needing to initiate or restart cancer treatment. ASCO and NICE have published generic guidance, while others (ESMO, French society) have a short comment, leaving the decision making to individual clinical evaluation. Urgent development is needed in: virus testing method and frequency, confirmatory tests, definition of adequate asymptomatic period, management of persisting COVID-19 positivity and evaluation of potential risk stratification factors.

One of the greatest challenges is how to advise cancer patients on COVID-19 risks when there is a lack of evidence in this matter. Though some patients may appreciate the clinician’s honesty, others may leave it to their doctor to make the judgement call (34).

We should not forget that, as with previous pandemics, COVID-19 may present subsequent infection peaks, influenced by factors such as seasonal and regional variation (35, 36). In the Northern Hemisphere countries are still heavily affected by the pandemic and any relaxation of the first phase stringent measures may result in a second surge within the coming months. Additionally, concerns are raised about COVID-19’s impact on the Southern Hemisphere countries that have limited medical and economic resources to counteract the threat.

As our understanding of COVID-19 grows, management approaches may be intensified, added, dropped or permanently implemented in our clinical practice. During data collection for this report we noticed changes in the available guidelines, and expect further evolution to a more permanent adapted model.

## Conclusions

Oncological societies have quickly developed and adapted recommendations in these unprecedented circumstances. As clinicians, we strive to provide the best cancer management and treatment in the face of COVID-19 uncertainties (37).

## Author Contributions

KK, DM, KP, AA, and MT: Conception or design of the work. NH, BP, MD, and AV: Data collection. PK, JK, CK, and LP: Data analysis and interpretation. NC, GZ, NP, CC, HS, FM, and IP: Drafting the article. RM, BM, JG, and DT: Critical revision of the article. KK, DM, KP, AA, RM, JG, DT, and MT: Final approval of the version to be published. All authors contributed to the article and approved the submitted version.

## Funding

All authors declare that the research was conducted in the absence of any commercial or financial relationships.

## Conflict of Interest

The authors declare that the research was conducted in the absence of any commercial or financial relationships that could be construed as a potential conflict of interest.

## Appendix

**Appendix Table 1:** Guidelines demographics across screened societies *

Country/Region	Society	Acronyms (Abbreviations)	Guidance
Australia	Medical Oncology Group of Australia	MOGA	Y
	Australian Government-Cancer Australia	AG-CA	Y
Austria	Austrian Society for Hematology and Medical Oncology	ÖGHO	Onkopedia
Belgium	Belgian Society of Medical Oncology	BSMO	Y
Brazil	Sociedade Brasileira de Oncologia Clínica	SBOC	Y
Bulgaria	Bulgarian National Association of Oncology	BNAO	–
	Bulgarian Association of Medical Oncology	BAMO	–
	Expert Council Panel		Y
Canada	Cancer Care Ontario	CCO	Y
	British Columbia Cancer	BCC	Y
China	Breast Cancer expert committee, National Cancer Quality control Center	NCQC-BC	Y
Croatia	Croatian Society for Medical Oncology	HDIO	Y
Cyprus	Cyprus Oncology Society	OEK	Y HeSMO, ASCO
Czech Republic	Czech Society for Oncology	CSO	Y
France	Haut Conseil de Santé Publique	HPSP	Y
Germany	Deutsche Gesellschaft für Hämatologie und Medizinische Onkologie	DGHO	Onkopedia
Greece	Hellenic Society of Medical Oncology	HESMO	Y
Italy	Italian Association of Medical Oncology	AIOM	Y
Japan	Japanese Society of Medical Oncology	JSMO	ASCO
Jordan	Jordanian Oncology Society	JOS	NCCN, ASCO
Netherlands	Nederlandse Vereniging voor Medische Oncologie	NVMO#	Y
Portugal	Sociedade Portuguesa de Oncologia	SPO	Y
Serbia	Serbian Society for Medical Oncology	UMOS	ESMO
Slovenia	Institute of Oncology Ljubljana	IOL^#^	Y
	Slovenian Society of Medical Oncology	SIO	ESMO, ASCO
Spain	Sociedad Española de Oncología Médica	SEOM	Y
Sweden	Swedish Society of Oncology—Svensk Onkologisk Förening	SOF#	Y
	Regional Cancer Centre in Cooperation	RCCC	Y
Switzerland	Swiss Society of Medical Oncology	SSMO·SSOM·SGMO	Onkopedia
UK	National institute for health and care excellence	NICE	Y
USA	American Society of Clinical Oncology	ASCO	Y
	National Comprehensive Cancer Network	NCCN	Y
Europe	European Society for Medical Oncology	ESMO	Y

Data cut-off for screening of National Societies and International Societies was April 30, 2020.

^#^Guidelines available only for members of the society

* Source of information provided through the publications/links below:

Australia:

file:///C:/Users/v101nv/Downloads/MOGA-Endorsed_Practical-considerations-for-the-management-of-cancer-patients-during-the-COVID19-pandemic-April-2020.pdf

https://www.mja.com.au/journal/2020/212/10/managing-haematology-and-oncology-patients-during-covid-19-pandemic-interim

Austria: www.onkopedia.com ; Medical Chamber: www.aerztekammer.at, Health Ministry: www.sozialministerium.at

Belgium: https://www.bsmo.be/covid-19-and-cancer/

Brazil: https://sboc.org.br/images/Infografico_-_Coronav%C3%ADrus_para_medicos_v5.pdf

Bulgaria: https://1drv.ms/u/s!ApvZAv8wL0KhrRhC0aoQhA94RkRJ?e=lnIDgU

Canada: http://g-o-c.org/wp-content/uploads/2020/04/OH-CCO-COVID-19-Pandemic-Planning-Supplemental-Guidance-Cancer-2020-03-29.pdf. https://www.accc-cancer.org/docs/documents/cancer-program-fundamentals/oh-cco-pandemic-planning-clinical-guideline_final_2020-03-10.pdf.

http://www.bccancer.bc.ca/health-professionals site/Documents/provincial_cancer_clinical_management_guidelines_pandemic_situation_covid19_april20_2020.pdf.

https://www.agreetrust.org/wp-content/uploads/2013/10/AGREE-II-Users-Manual-and-23-item-Instrument_2009_UPDATE_2013.pdf.

China: http://books.ipmph.com/books/detail/2036483.shtml

Croatia: http://www.internistickaonkologija.hr/wp-content/uploads/2020/04/HDIO-djelatnici_COVID-19.pdf

Czech Republic: https://www.linkos.cz/ceska-onkologicka-spolecnost-cls-jep/organizace-cos/vyjadreni-vyboru-cos-cls-jep-k-situaci-souvisejici-se-sirenim-koronaviroveho-one/

National Governance:  https://koronavirus.mzcr.cz/informace-pro-zdravotniky/

Cyprus: https://oncology-cy.eu/

France: You B, Ravaud A, Canivet A, et al. The official French guidelines to protect patients with cancer against SARS-CoV-2 infection. Lancet Oncol. 2020 May;21(5):619-621

Germany, Switzerland: www.onkopedia.com

Greece: https://mcusercontent.com/a1de5b637ce118da45457bd62/files/cf3a4436-0134-45a2-a04c-24eabd8e8b93/%CE%9F%CE%B4%CE%B7%CE%B3%CE%B9_%CE%B5%CF%82_%CE%B1%CE%BD%CF%84%CE%B9%CE%BC%CE%B5%CF%84%CF%89_%CF%80%CE%B9%CF%83%CE%B7%CF%82_%CE%B1%CE%B9%CE%BC%CE%B1%CF%84%CE%BF%CE%BB%CE%BF%CE%B3%CE%B9%CE%BA%CF%89_%CE%BD_%CE%BA%CE%B1%CE%B9_%CE%BF%CE%B3%CE%BA%CE%BF%CE%BB%CE%BF%CE%B3%CE%B9%CE%BA%CF%89_%CE%BD_%CE%B1%CF%83%CE%B8%CE%B5%CE%BD%CF%89_%CE%BD_%CE%BC%CE%B5_COVID_19.pdf

Italy: https://www.aiom.it/wp-content/uploads/2020/03/20200313_COVID-19_indicazioni_AIOM-CIPOMO-COMU.pdf

Spain: https://seom.org

Sweden: https://www.cancercentrum.se/samverkan/covid-19/avvikelser-i-behandlingsrekommendationer/(RCCC)

Portugal: https://www.sponcologia.pt/fotos/editor2/publicacoes/recomendacoes_para_o_tratamento.pdf

UK: https://www.nice.org.uk/guidance/ng161

US: https://www.asco.org/asco-coronavirus-information

https://www.nccn.org/covid-19/

Europe: https://www.esmo.org/guidelines/cancer-patient-management-during-the-covid-19-pandemic

## Appendix

**Appendix Table 2:** Selected key points for Service Delivery.

	Set/follow management plans	Staff training	Phone/Video Consultation	Postpone non-essential follow-up visits	“Previous day” phone triage	Checkpoint areas	Triage before OP	Reduce timein Ops		Waiting area/Distance between pts
**Australia** ***MOGA, AG-CA***	Y	Y	Y	Y	Y	Y	Y	Y		Y
**Austria** ***Onkopedia, AHM, AMC***	Y	Y (AHM)	Y (AMC)	Y	–	Y (AHM)	Y ((AHM)	Y (AMC)		Y (AMC)
**Belgium** ***BSMO***	–	–	Y	Y	–	–	Y	–		–
**Brasil *SBOC***	Y	–	Y	–	–	–	Y	–		Y
**Bulgaria** **Expert Council Panel**	Y	Y	Y	Y	Y	Y	Y	Y		Y
**Canada CCO**	Y	–	Y	Y	Y	–	Y	Y		–
**Canada BCC**	Y	Y	Y	Y	Y	Y	Y	–		–
**China**	Y	Y	Y	Y	Y	Y	Y	Y		Y
**Croatia** ***HDIO***	–		Y	Y	Y	Y	Y	Y		Y
**Cyprus** ***OEK***	–	Y	Y	Y	–	Y	Y	Y		Y
**Czech Republic** ***CSO*** ***National governance****	Y	Y *	Y*	Y	Y *	Y*	Y*	Y*		Y*
**France** ***HPSP***	–	–	Y	–	–	–	–	–		Y
**Germany/Switzerland** ***Oncopedia***	–	–	Y	–	–	–		–		–
**Greece *HESMO***	Y	Y	Y	Y	Y	–	Y	Y		Y
**Italy *AIOM***	Y (for high risk regions)	Y	Y	Y		Y	Y			Y
**Netherlands *NVMO***			Y	Y	Y		Y			
**Portugal *SPO***	Y	Y	Y	Y	Y	–	Y	–		Y
**Slovenia *IOL***	Y	Y	Y	Y	Y	Y	Y	Y		Y
**Spain *SEOM***	Y	–	Y	Y	–	–	–	Y		Y
**Sweden SOF, RCCC**	Y	Y	Y	Y	–	–	–	–		–
**UK *NICE***	Y	Y	Y	Y	–	–	–	Y		–
**USA** ***ASCO*** ***NCCN*** ***CDC***	–	Y	Y	Y	Y	Y	Y	–		Y
**Europe** ***ESMO***	–	–	Y	y	Y	Y#	Y#	Y#		Y#
**Country/Medical Society**	**Home Blood sampling**	**Home delivery of oral Meds/** **iv infusion**	**Contact infected pt regularly**	**Service provision from self-isolated/infected staff**	**If Cancer Centre is COVID affected**	**Work in Groups/** **Set up shifts** **Suspend shifts**	**Web use by HCPs**	**Psychological support for HPs**		**HCPs to monitor their T**
**Australia** ***MOGA, AG-CA***	Y	Y	–	–	–	–	Y	–		–
**Austria** ***Onkopedia, AHM, AMC***	–	–	–	–	–	–	–	Y (AHM)		–
**Belgium *BSMO***		Y								
**Brasil** ***SBC***	–	–	–	–	–	–	–	–		–
**Bulgaria** **Expert Council Panel**	–	–	*Y*	–	Y	Y	Y	*Y*		–
**Canada *CCO***	–	Y	–	–	–	–	–	–		–
**Canada BCCA**	–	Y	–	–	–	–	–	–		–
**China**	Y	Y	Y	Y	Y	Y	Y	Y		Y
**Croatia** ***HDIO***	–	–	–	–	–	Y	Y	–		Y
**Cyprus** ***OEK***	Y	Y	–	–	–	Y	–	–		Y
**Czech Republic** ***CSO; National governance****	–	–	–	Y *	Y*	Y*	–	–		Y*
**France** ***HPSP***	–	Y	–	–	–	–	–	–		–
**Germany/Switzerland** ***Oncopedia***		–	–	–	–	–	–	–		–
**Greece** ***HESMO***	Y	Y	–	–	Y	Y	–	–		-
**Italy** ***AIOM***	–	–	–	–	–	–	–	Y		–
**Netherlands *NVMO***	Y	Y	Y(48 hours)	–	–	–	–	–		–
**Portugal** ***SPO***,	Y	Y	Y	Y	Y	Y	–	Y		Y
**Slovenia** ***OIL, SIO***	Y	–	–	Y	Y	Y	Y	–		Y
**Spain** ***SEOM***	–	Y	–	–	Y	–	–	–		–
**Sweden** **SOF, RCCC**	–	–	–	–		–	–	Y		–
**UK** ***NHS,NICE***	–	Y	daily	Y	–	–	–	–		–
**USA** ***ASCO*** ***NCCN*** ***ACS***	Y	Y	Y	Y	Break/use other units	–	–	Y		Y(referral to CDC recommendation)
**Europe** ***ESMO***	Close to Home	Y	–	–	–	Y	Y	Y		–
**Country/Medical Society**	**Oncology Hospital Meetings**		**HCPs to minimize hospital Circulation and surface contact**		**Non-COVID** **Oncology circuits to be formed**		**Invasive Ventilation**			**Radiological Imaging**
**Australia** ***MOGA, AG-CA***	Y		–		Y		–			–
**Austria** ***DGHO***	MDT (video/phone)		–		–		–			–
**Belgium *BSMO***	Y									
**Brasil** ***SBC***	–		–		–		–			–
**Bulgaria** **Expert Council Panel**	Y		–		–		–			–
**Canada *CCO***	–		–		–		Y			Y
**Canada BCC**										
**China**	Y									
**Croatia** ***HDIO***	Y MDT avoid contacts, tele		Y		–		–			Delay
**Cyprus** ***OEK***	MDT teleconference		Y							Postpone routine scans/tests
**Czech Republic** ***CSO***	–		–		–		–			Y, can be delayed in specified groups
**France** ***HPSP***	–		–		–		–			
**Germany/Switzerland** ***Oncopedia***	–		–		–		–			–
**Greece** ***HESMO***	Y		–		–		–			Delay
**Italy** ***AIOM***	–		–		–		–			–
**Japan** ***JSMO***	MDT Minimize contacts				Y					Delay
**Jordan** ***JRMS***	MDT Minimize contacts				Y					Delay
**Netherlands** ***NVMO***	MDT Minimize contacts		–		–		Y			Delay
**Portugal** ***SPO, SPRO***	MDT Video		Y		Y		Y			–
**Slovenia** ***IOL***	Y (only MTD with limited stuff)		Y		Y		Y (we have in readiness a COVID department with ventilators)			Y (reduced and without peroral contrast to reduce time spent in hospital)
**Spain** ***SEOM***	Y (MDT by videoconference)		–		–		Y Individualization (see the Ethics Document)			Y: Individualization
**Sweden** **SOF, RCCC**										Y (individualization)
**UK** ***NHS, NICE***	–		–		–		–			–
**USA** ***ASCO*** ***NCCN*** ***ACS***	–		–		y		Y:Ref to POLST			Y
**Europe** ***ESMO***	–		–		–		–			Y (according to priority group)

AHM, Austrian Health Ministry; AMC, Austrian Medical Chamber; esp., especially; pt, patient; PPE, Personal Protective Equipment; resp., respiratory; CDC, Centers for Disease Control and Prevention; HCP, Health Care Professional; T, Temperature; OP, Outpatient clinics.

Y*: issued by national guide and adopted by oncology society

“Y^#^”: presented as an option applied in several institutions but not as a recommendation in paragraph: “Additional prevention measures in hospitals and health centers”.

## Appendix

**Appendix Table 3:** Selected key points for General Treatment Measures.

Selected key points for General Treatment Measures													
	Treatment initiation	Treatment Prioritization	GCSF/Abx use	Supportive Tx, e.g., BPhs/ BT	Treatment breaks/delays	Change iv to sc/oral Tx	Decrease frequency of Tx	Shorter regimes	Longer treatment supplies	Suspend/minimize doses	Consent for COVID	Stratified follow-up models	Post-COVID Tx
**Australia** ***MOGA, AG-CA***	Y	Y	Y		Y	Y	Y	Y	–	Y	Y	Y	–
**Austria**, **Germany** **Switzerland** ***Oncopedia***	Y (individualized)	–	–	Y (BT)	Y (individualized)	–	–	–	–	Y (ISs if appropriate)	–	–	–
**Belgium *BSMO***	Delay if appropriate	–	–	–	–	–	–	Y (Adj)	–	–	–	–	–
**Brasil** ***SBOC***	–	–	–	–	–	–	–	–	–	–	–	–	–
**Bulgaria** **Expert Council Panel**	Y	–	Y	Y	Y	Y	Y	–	–	–	–	Y	–
**Canada** ***CAMO-ACOM*** ***CCS***	–	–	–	–	–	–	–	–	–	–	–	–	–
**China**	Y	Y	Y		Y	Y	Y	Y	Y		Y	Y	–
**Croatia** ***HDIO***	Y	Y	Y	Y	Y	Y	Y	Y	Y	Y	–	Y	–
**Cyprus** ***OEK***	Y, Delay if appropriate	Y	Y	–	Y	Y	Y	Y	Y	–	–	–	–
**Czech Republic** ***CSO***	Y, Delay if appropriate	Y	–	–	Y	Y	Y	Y	Y	Y	–	Y	–
**France** ***HPSP***	Y	Y	–	–	Y	Y	Y	–	–	–	–	–	Recovered Pt
**Greece** ***HESMO***	Y	Y	Y	BT	Y	Y	Y (ITx)	–	–	Y	–	–	Y
**Italy** ***AIOM***	Delay if appropriate	–	–	–	Y	Y	–	–	–	–	–	–	–
**Netherlands** ***NVMO***	Postpone + cases		Against	–	Y in asymptomatic	–	–	–	Y	Dose reductions	–	–	–
**Portugal** ***SPO***	Y	Y	–	Y	Y	Y	Y	Y	Y	Y	Y	Y	–
**Slovenia** ***IOL***	Y	Y	Y	Y	Y (for non-curative only)	Y	Y for non-curative)	Y	Y	optional	–	Y	–
**Spain** ***SEOM***	Do not start in infected cases Consider delays	Y	Y	Y (consider delay)	Y	Y	Y	Y	Y (for oral drugs)	Steroids (low or high dose) ISs (e.g., everolimus)	Y	Y	Y (recovered patient)
**Sweden** **SOF, RCCC**	Y	Y	Y	Y	Y	Y	Y	Y	–	Y	–	Y	–
**UK** ***NHS,NICE***	–	Y	Y	Defer BPhs unless for Hyper Ca	Y	Y	Y (ITx)	Y	Y	–	–	Y	One negative test
**USA** ***ASCO, NCCN*** ***ACS, CDC***	y	y	Y	y	Y	Y	y	y	y	y	Y (documented IC)	–	Symptom free^ASCO, CDC^ Neg test^ASCO^ 2neg tests^CDC^
**Europe** ***ESMO***	RBA discussion	Y	y	BT when strictly necessary	Y	Y	Y	Y for RT	Y 3 courses for oral Tx	Y	–	Y esp.in lung and senior pts	Y but no specific guide ,” to discuss with patient”

Y, Yes; -, Not available; GCSF, Granulocyte Colony stimulating factor; ABx, Antibiotics; Tx, Treatment; BPhs, Bisphosphonates; BT, Blood Transfusion; i.v., intravenous; sc, subcutaneous; ITx, Immunotherapy; Iss, Immunosuppressants; Adj, Adjuvant; NR, Not Recommended; RBA, Risk-Benefit Assessment.

## Appendix

**Appendix Table 4:** Personal and Patients’ protective face mask.

	Face mask/antiseptic for staff	Type of Face mask for staff	Face mask/ antiseptic for pts	Type of Face mask for patients
**Australia** ***MOGA, AG-CA***	Y	SM Consider respirators in high-risk pts.	Y	SM
**Austria** ***Onkopedia, AHM, AMC***	Y (AHM)	1) SM or FFP2. 2) Suspected or confirmed COVID-19: PPE+FFP2.	Y	SM or FF2
**Belgium** ***BSMO***	Y	No specific mask type.	Y	No specific mask type.
**Brasil *SBOC***	Y	N/A	Y	N/A
**Bulgaria** **Expert Council Panel**	*Y*	*SM or N95 or FFP2/3*.	–	*SM or textile mask*.
**Canada CCO**	Y	1) Staff who treats COVID-19 + pts who or "high-risk" pts for virus transmission: personal protective equipment.	–	–
**Canada BCC**	Y	2) For high-risk of aerosolization procedures: Consider performing a low-risk procedure. If not possible, use N95 mask.	–	–
**China**	Y	N/A	Y	N/A
**Croatia** ***HDIO***	Y	1) SM 2) Suspected or confirmed COVID-19: FFP2 respirator. 3) Suspected or confirmed COVID-19, for aerosol generating procedures: FFP3 respirator.	Y	1) SM or textile mask). 2) Suspected or confirmed COVID-19: SM.
**Cyprus** ***OEK***	Y	SM	Y	SM
**Czech Republic** ***CSO*** ***National governance****	Y*	1) SM. Tend to use FFP2. 2) Suspect COVID-19: FFP2 respirators, tend to use FFP2. 3) Confirmed COVID-19: FFP2 respirators.	Y*	1) No COVID-19 signs: SM 2) Suspect COVID-19: SM. 3) Confirmed COVID-19: SM
**France** ***HPSP***	Y	SM	Y	SM
**Germany/Switzerland** ***Oncopedia***	Y	1) SM or FFP2. 2) Suspected or confirmed COVID-19: PPE+FFP2.	Y	SM or FF2
**Greece *HESMO***	Y	SM	Y	SM
**Italy *AIOM***	Y	SM Where available FFP2-FFP3 mask.	Y	SM
**Netherlands *NVMO***	Y	No specific mask type.	Y	No specific mask type
**Portugal *SPO***	Y	N/A	Y	N/A
**Slovenia *IOL***	Y	a) Contact with COVID+ pts: IIR in combination with visir. b) Intubation of surgical COVID-pts, <24 hours: mask FFP2+visir. c) Intubation >;24 hours COVID-pts : mask FFP3+visir.	Y	SM
**Spain *SEOM***	Y	SM	Y	Immunosuppressed pts: FFP2 masks, with no valve.
**Sweden SOF, RCCC**	Y (in assessment of patients with COVID-19 symptoms).	No specific mask type.	–	No specific mask type.
**UK *NICE***	Y	SM	Y	SM
**USA** ***ASCO*** ***NCCN*** ***CDC***	Y (CDC)	1) SM + eye covering. 2) Suspected or confirmed COVID-19: n95.	Y	SM or textile mask
**Europe** ***ESMO***	–	SM	Y	SM

SM, Surgical Mask, pts, patients; N/A, Not available.
